# IgG4 Related disease – a retrospective descriptive study highlighting Canadian experiences in diagnosis and management

**DOI:** 10.1186/1471-230X-13-168

**Published:** 2013-12-09

**Authors:** Harshna Patel, Korosh Khalili, Kim Tae Kyoung, Leyla Yazdi, Eric Lee, Gary May, Paul Kortan, Catalina Coltescu, Gideon M Hirschfield

**Affiliations:** 1 Liver Centre, Toronto Western Hospital, University of Torontocpf, Toronto, ON, Canada; 2 The Scarborough Hospital, Scarborough, ON, Canada; 3 Department of Medical Imaging, University Health Network, Toronto, ON, Canada; 4 Centre for Advanced Therapeutic Endoscopy and Endoscopic Oncology, St Michael’s Hospital, Toronto, ON, Canada; 5 Centre for Liver Research and NIHR Biomedical Research Unit, Institute of Biomedical Research, The Medical School, University of Birmingham, 5th Floor, Birmingham B15 2TT, UK

**Keywords:** Autoimmune pancreatitis, Sclerosing cholangitis, IgG4

## Abstract

**Background:**

Appreciating the utility of published diagnostic criteria for autoimmune pancreatitis, when compared to the characteristics of patients clinically managed as having disease, informs and refines ongoing clinical practice.

**Methods:**

Comparative retrospective descriptive evaluation of patients with autoimmune pancreatitis including dedicated radiology review.

**Results:**

66 subjects with radiographic OR clinical features of autoimmune pancreatitis were initially identifiable (Male: n = 50), with 55 confirmed for evaluation. The most common presentation included pain (67%), weight loss (65%), and jaundice (62%). Diffuse enlargement of the pancreas was evident in 38%, whilst multifocal, focal, or atrophic changes were seen in 7%, 33% and 9% respectively. 13% had no pancreatic parenchymal involvement. Peripheral rim enhancement was seen in 23 patients (42%). Where discernible, disease was a) Sclerosing pancreatitis and cholangitis, n = 21; b) Sclerosing cholangitis, n = 9; c) Sclerosing pancreatitis, n = 4; d) Sclerosing pancreatitis and cholangitis with pancreatic pseudotumour, n = 7; e) Sclerosing cholangitis with hepatic pseudotumour, n = 3; f) Sclerosing pancreatitis with pancreatic pseudotumour, n = 1. 56% of the patients had systemic manifestations and the median serum IgG4 at diagnosis was 5.12 g/L. The Korean criteria identified most patients (82%) compared to HISORt (55%) or the Japan Pancreas Society (56%). The majority (HISORt 60%; Japan Pancreas Society 55%; Korean 58%) met diagnostic criterion by radiological findings and elevated serum IgG4. Treatment and response did not differ when stratified by diagnostic criteria.

**Conclusion:**

Our descriptive and retrospective dataset confirms that in non-expert practice settings, autoimmune pancreatitis scoring systems with a focus on radiology and serology capture most patients who are clinically felt to have disease.

## Background

Autoimmune pancreatitis (AIP) is the umbrella term for a group of multi-system infiltrative and inflammatory relapsing and remitting conditions, not the least limited to pancreatic involvement, and for which clinical presentation is protean, and in which no single test is diagnostic [1-3]. Over the last decade much interest has been shown in this disease, with multiple descriptions of case series presented. This has been driven in part by increasing recognition of radiologic features of disease (such as irregular narrowing of the main pancreatic duct and enlargement of the pancreas), characteristic but not universal elevations of IgG4 levels, and lymphoplasmacytic infiltrates with abundant IgG4-positive plasma cells on immunostaining.

AIP is reported as more typically occurring in men (M:F ratio of 5:1) with an average age at presentation over 60. In Japan, where the predominant bulk of the experience of managing this disease arose, an estimated prevalence of 2.4 per 100,000 individuals is cited [4] with this disease accounting for about 6% of chronic pancreatitis, whilst in North America, ~2.5% of Whipple’s procedures performed for presumed pancreatic cancer are subsequently re-diagnosed as AIP and 20% of Whipple’s procedures for benign conditions are now considered likely to be AIP [5]. Presenting as a multisystem fibro-inflammatory condition there are various distinctive clinical, radiological, serological and pathological features, which point towards the diagnosis, particularly since no single uniform presentation predominates [6,7]. Frequently the diagnosis is reached in patients with painless obstructive jaundice secondary to an inflammatory pancreatic mass with biliary involvement. Abdominal pain and weight loss may also be present alongside exocrine or endocrine pancreatic insufficiency, whilst others present with extra-pancreatic disease: sclerosing cholecystitis, retroperitoneal fibrosis, sclerosing sialadenitis, sclerosing dacryoadenitis, interstitial nephritis, pulmonary interstitial fibrosis, lymphadenopathy, and pseudotumours. Extra-pancreatic disease is now recognized in 40–90% of patients with AIP and can be synchronous or metachronous [8,9]. The exquisite sensitivity of AIP to steroid therapy is a key feature in differentiating AIP from alternative processes [10], with clear clinical response to steroids usually striking, but disease relapse not infrequent upon steroid-withdrawal [11]. The repeated demonstration of elevated IgG4 levels in patients with PSC has also raised the spectre of a possible sub-group of patients with PSC who might have a forme-fruste or missed diagnosis of AIP, and steroids in this setting has been discussed widely [12,13].

Given such clinical heterogeneity there have arisen several potential diagnostic criteria available to clinicians to use in attempting to reach a diagnosis of AIP, and so facilitate early treatment. Each relies on a varied combination of imaging findings of the pancreas and other organs, serology, pancreatic histology and response to steroids. Their applicability in centres without dedicated pancreatic multi-disciplinary teams remains to be clarified, along with practical utility globally remaining under reported, particularly given that clinical and academic practice may not match. We present our descriptive retrospective review of patient presentation and outcomes for those with IgG4 related disease, seen across academic institutions in Toronto. In so doing we simultaneously document a Canadian experience of a large unselected cohort of patients with AIP, as well as reporting the applicability of selected diagnostic criteria in a North American setting.

## Methods

We evaluated the clinical, serologic, imaging characteristics and treatment response of patients given the clinical label of AIP from 1998 to 2010 across two sites within the Division of Gastroenterology at the University of Toronto: University Health Network and the Centre for Advanced Therapeutic Endoscopy and Endoscopic Oncology, St. Michael’s Hospital, Toronto, Canada. Approval for retrospective evaluation was obtained from University Health Network. Given the long time frame of review, and the changing appreciation of disease itself by clinicians, we acknowledge the limitations this places on the study, and therefore highlight the descriptive data retrieved.

**Figure 1:**
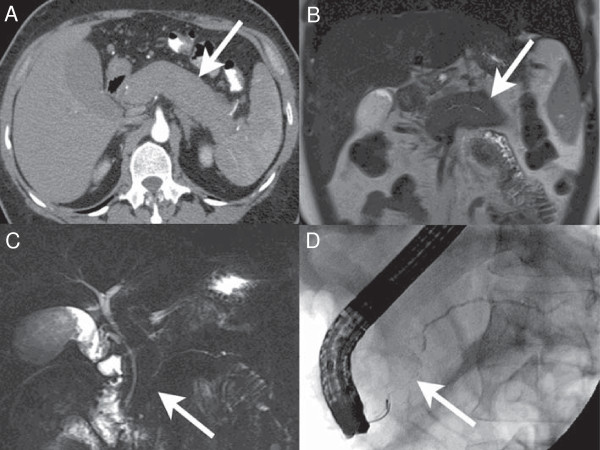
**Diffuse autoimmune pancreatitis. A**. Axial CT image in the pancreatic parenchymal phase shows the typical enlarged, poorly enhancing gland (arrow). Note the lack of inflammatory change around the organ which differentiates the disease from acute pancreatitis with necrosis. **B**. Coronal T2 Weighted MR image demonstrates low signal intensity in the pancreas (arrow) due to the diffuse fibrosis in the gland. **C**. Coronal MRCP image depicts a diffusely irregular pancreatic duct with stenosis distally in the pancreatic head (arrow). **D**. ERCP confirms the MR findings including the ductal stenosis (arrow).

**Figure 2:**
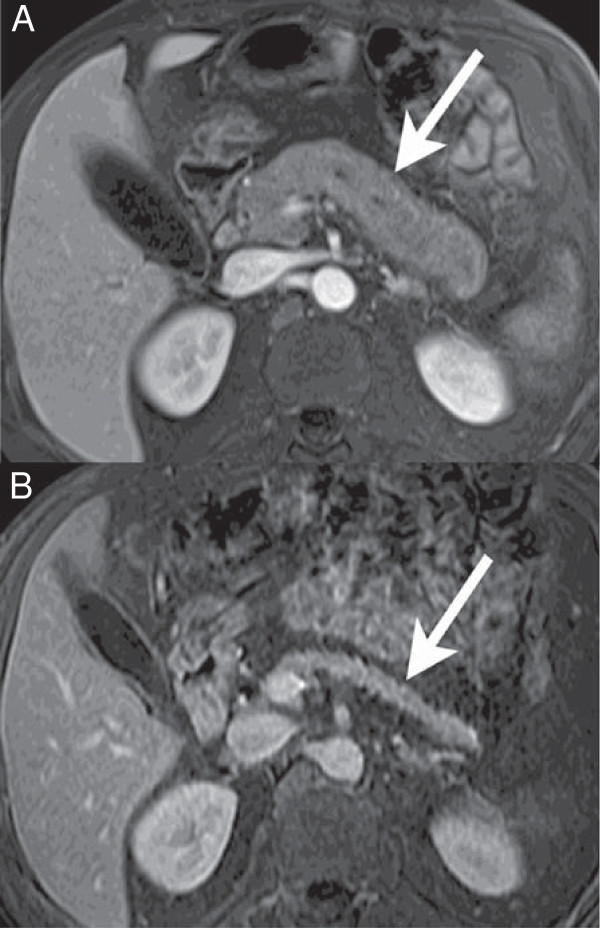
**Post-therapeutic pancreatic atrophy. A**. Axial contrast-enhanced T1 weighted MR image through the pancreas depicts an enlarged organ (arrow) with poor enhancement. Irregular beading of the pancreatic duct can be seen even on this image. **B**. Follow-up scan at the same level 12 months post steroid therapy shows marked atrophy of the pancreas (arrow) with normal enhancement.

A review of clinical notes (HP) and imaging studies was undertaken, followed by analyses of clinical and treatment outcomes. Two fellowship trained abdominal radiologists reviewed radiological findings in consensus and confirmed the radiological diagnosis of AIP. Imaging features reviewed included presence or absence of intra-hepatic and extra-hepatic biliary strictures as well as imaging abnormalities of pancreatitis. Imaging characteristics for each of the HISORt criteria [7], Japanese Pancreas Society [14] and Korean criteria [15] were also recorded (Figures 1 and 2; Additional file 1: Table S1). Imaging reports also included the anatomic involvement of strictures and inflammatory pseudotumours as originally described by Zen et al. [16]. If histological features were available from surgical resection or core biopsies, in particular lymphoplasmacytic infiltrate and positive IgG4 staining, then the diagnosis was definitive. In the absence of this, the diagnosis was also made if patients had classical radiological features of AIP (diffusely enlarged pancreas with delayed enhancement and capsule-like rim; diffusely irregular, attenuated main pancreatic duct or multiple strictures or long stricture without upstream dilatation) and an elevated serum IgG4 > 2 × upper limit of normal (ULN) (>1.72 g/L). Patients not meeting either of these two criteria but in whom a high index of suspicion existed (elevated serum IgG4, suggestive pancreatic imaging findings, other organ involvement or bile duct biopsy with >10 IgG4 positive cells per high power field) would undergo a trial of steroid therapy, and a positive clinical response was considered diagnostic. Malignancy at initial presentation was excluded in all cases. Given our selection criteria we report on type 1 autoimmune pancreatitis; the relative importance of type 2 autoimmune pancreatitis (so called idiopathic duct-centric pancreatitis; those with no IgG4 and no lymphoplasmocytic infiltrates but with granulocyte epithelial lesions, venulitis and storioform fibrosis) cannot be adequately addressed by our study; in our chart survey only 4 patients were provisionally identified that were potentially type 2 AIP and only limited clinical information on 2 was available, hence our focus being classic AIP.

Our series represents the standard clinical practice over the duration of the study period, with no single treatment protocol, and many treating physicians. Treatment regimens early tended to be heterogeneous, reflecting clinical judgement and the evolving knowledge and confidence in managing this condition. A typical treatment approach for active disease involved the commencement of prednisone at a dose of 40 mg daily, with a gradual taper over ~ three months’ duration depending on an assessment of clinical response. Patients who had clinically significant biliary obstruction underwent therapeutic endoscopic retrograde cholangiopancreatography (ERCP), with re-assessment of the biliary stricture(s) on follow-up ERCPs undertaken and intervention based on repeat cholangiography. Routine clinical review and monitoring (including serum IgG4) were performed and evidence of clinical, biochemical or serologic disease-relapse was sought as part of clinical follow-up. Response to steroid therapy was considered if there was a significant improvement in liver biochemistry and/or imaging. Clinical remission was defined as achieving a steroid-free state, with resolution of biliary stricture(s) (stent-free) and previously abnormal liver biochemistry.

## Results

### Patient demographics and clinical presentation

**Figure 3:**
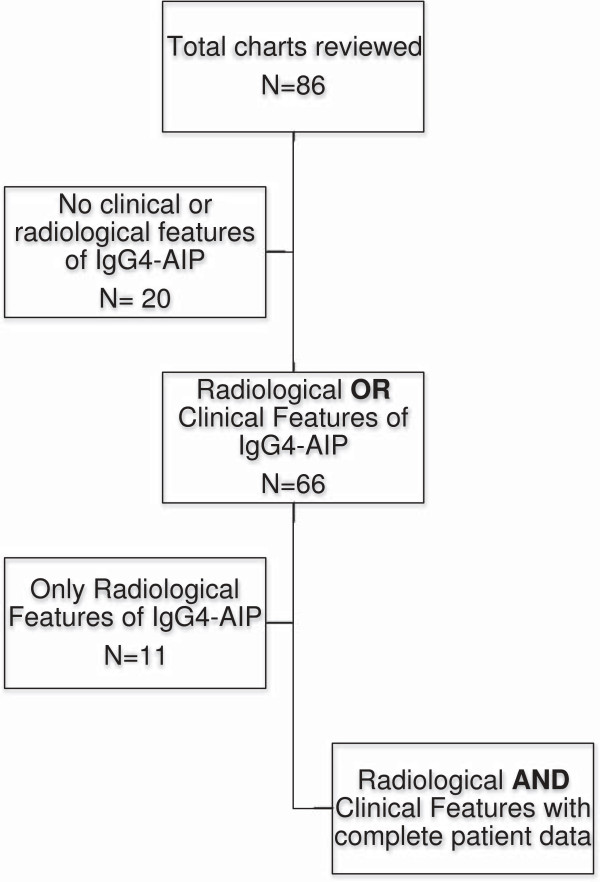
Study design and patient evaluation.

A total of eighty-six patients considered as potential AIP were first screened looking for sufficient clinical information for inclusion in the study (see study flow chart, Figure 3). Sixty-six patients with comprehensive data had radiographic changes *OR* clinical features of autoimmune pancreatitis (Male 50, Female 16; mean age at diagnosis 58) (Table 1). Of these, eleven patients had radiological reports available but no clinical data charted within our Institutions leading to exclusion from further study. Thus, a total of fifty-five patients with a combination of clinical and/or radiological features of AIP were identified, with a predominance of men (78%) and mean age at diagnosis of 60.

**Table 1:** Patient demographics

**Findings**	**Total study**	**All criteria**	**HISORt**	**Japanese**	**Korean**	**No criteria met**
Radiological features	66	-	-	-	-	-
Males (n, %)	50 (76%)	-	-	-	-	-
Age (years, mean)	58	-	-	-	-	-
Clinical and radiological features	55	27 (49%)	30 (55%)	31 (56%)	45 (82%)	9 (16%)
Males (n, %)	43 (78%)	22 (81%)	25 (83%)	24 (77%)	34 (76%)	8 (%)
Age (years, mean)	60	61	61	59	60	58
Presenting symptoms						
Jaundice	34 (62%)	20 (74%)	22 (73%)	20 (65%)	29 (64%)	5 (56%)
Weight loss	36 (65%)	17 (63%)	19 (63%)	19 (61%)	30 (67%)	6 (67%)
Abdominal pain	37 (67%)	18 (67%)	20 (67%)	20 (65%)	29 (64%)	7 (78%)
IgG4 (g/L, median)	5.12	5.32	5.44	5.3	4.94	7.3
IgG4 > 2 × ULN (n, %)	29 (51%)	20 (74%)	21 (70%)	24 (80%)	24 (53%)	4 (44%)
Biochemistry						
Bilirubin (μmol/L*)	20	35	34	34	21	29
ALP (U/L*)	246	287	287	264	240	296
ALT (U/L*)	74	94	94	81	72	91
AST (U/L*)	57	92	92	84	58	46
Imaging Modality						
CT (n, %)	49 (86%)	23 (85%)	26 (87%)	29 (84%)	40 (89%)	8 (89%)
MRCP (n, %)	54 (95%)	27 (100%)	30 (100%)	31 (100%)	44 (98%)	9 (100%)
ERCP (n, %)	35 (61%)	19 (70%)	22 (73%)	19 (61%)	28 (62%)	6 (67%)
Pancreatic parenchymal changes						
Typical	24 (44%)	14 (52%)	14 (47%)	14 (45%)	20 (44%)	4 (57%)
Diffuse	5 (9%)	3 (19%)	4 (13%)	3 (10%)	4 (9%)	0 (0%)
Multifocal	23 (42%)	11 (41%)	11 (37%)	13 (42%)	18 (40%)	5 (56%)
Rim						
Atypical	18 (33%)	5 (19%)	7 (23%)	9 (29%)	16 (36%)	2 (22%)
Focal	5 (9%)	4 (15%)	5 (17%)	4 (13%)	5 (11%)	0 (0%)
Atrophic						
Treatment						
Steroids	34 (62%)	19 (70%)	21 (70%)	22 (71%)	31 (69%)	3 (33%)
ERCP + Biliary stent	27 (49%)	15 (56%)	17 (57%)	15 (48%)	23 (51%)	4 (44%)
Steroids and biliary stent	16 (29%)	10 (37%)	12 (40%)	10 (32%)	16 (36%)	0 (0%)

*Median.

Whilst the majority of patients were Caucasian (55%), significant numbers of non-Caucasian patients were seen: 25% South Asian, 18% East/South-East Asian, 2% African, and 2% Middle Eastern. The mean follow-up from diagnosis was 22 months and the median time from symptom onset to definitive diagnosis of AIP was 8 months (max 98 months). 10 patients underwent major surgery for suspected malignancy prior to a definitive diagnosis of AIP. The most common clinical features at presentation were abdominal pain (67%), weight loss (65%), and obstructive jaundice (62%) and 34 of 55 patients had 3 or more symptoms, including fatigue (35%), new-onset diabetes mellitus (22%), pruritus (22%), and steatorrhoea (18%). One patient was subsequently diagnosed with cholangiocarcinoma and was the only patient with disease-associated mortality.

### Imaging

Pre-treatment cholangiography and/or pancreatic imaging at diagnosis were performed for all patients and a dedicated radiologist reviewed all images. Fifty-four patients (98%) had magnetic resonance cholangiopancreatography (MRCP) while 35 patients (64%) also underwent ERCP, the main indication being obstructive jaundice (29 of 35 patients). Diffuse enlargement of the pancreas was seen in 38% of patients followed for AIP, whilst multifocal, focal, atrophic changes were seen in 7%, 33% and 9% of patients respectively. Thirteen percent had no pancreatic parenchymal involvement. Peripheral rim enhancement was seen in 23 patients (42%). The fibro-inflammatory process affected both the biliary tree and pancreas in the majority of patients. Where discernible, disease was a) Sclerosing pancreatitis and cholangitis, n = 21; b) Sclerosing cholangitis, n = 9; c) Sclerosing pancreatitis, n = 4; d) Sclerosing pancreatitis and cholangitis with pancreatic pseudotumour, n = 7; e) Sclerosing cholangitis with hepatic pseudotumour, n = 3; f) Sclerosing pancreatitis with pancreatic pseudotumour, n = 1. Overall, intra-hepatic (65%) and extra-hepatic (76%) biliary strictures, and imaging abnormalities of pancreatitis (62%) were evident in most patients.

### Extra-pancreatic manifestations

In keeping with the multi-systemic nature of this disease process, 56% (n = 33) of the patients had systemic manifestations, including generalized lymphadenopathy (n = 15), renal involvement (n = 14), retroperitoneal fibrosis (n = 9), salivary gland enlargement (n = 6), inflammatory bowel disease (IBD) (n = 4), and lung involvement (n = 2). Thirteen patients had involvement of two or more extra-pancreatic organs. All extra-intestinal manifestations except IBD were made by radiological changes.

### Pre-treatment serology

38/55 (69%) patients had serum IgG4 levels accessible to our review. Of these 38, 29 (81%) had IgG4 levels twice the normal limit. The median serum IgG4 at diagnosis for our series was 5.12 g/L (range 0.01-20.00; normal value <0.86) falling to 1.5 g/L (n = 38; range 0.08-16.7) after treatment. An abnormality in the liver biochemistry was seen for the majority of patients at the time of presentation (69%): bilirubin 20 μmol/L (median, range 5–407), ALP 246 U/L (median, range 47–1226), ALT 74 U/L (median, range 14–784), and AST 57 U/L (median, range 13–542).

### Histology

IgG4-staining was not routinely offered by the Institutional laboratories and routine tissue sampling not performed. Histological (surgical) evidence of a lymphoplasmacytic infiltrate with sclerosis was available for seven patients.

### Steroid treatment and response

Thirty-four patients (62%) had sufficient ongoing clinical disease to receive steroid therapy following diagnosis. Thirty-five (64%) patients underwent ERCP at diagnosis of which 27 needed biliary stenting, and 16 (29%) patients went on to receive concurrent steroid therapy and biliary stenting. Eighteen patients were treated with steroids alone. Liver biochemistry (serum bilirubin, alkaline phosphatase and transaminases) normalized in ten of the eighteen patients with steroid monotherapy, improved in 2 (fell but not to normal) and did not change in 6 patients. Of the eleven patients who only underwent biliary stenting, liver enzymes normalized in 6, improved in 2 and did not change in 3 patients. In the 16 patients who received steroids and biliary stenting, liver enzymes became normal in 11 patients and improved in 5 patients. However, stricture resolution was achieved in only 3 of 18 patients in the steroid only group, in 6 of the 11 who underwent biliary stenting, and in 8 of 16 patients who received both steroids and biliary stenting.

### Disease relapse

Steroids were tapered and completely withdrawn in 20 of the 34 patients after a median exposure to steroids of 8.1 months. Disease-relapse occurred in 15 patients after a median of 15 month necessitating the resumption of steroids and addition of an immunomodulator (azathioprine) in 14 patients. Two patients received Rituximab [17]. Both had steroid intolerance (infection; diabetes).

### Stratification by diagnostic criteria

Published diagnostic criteria were applied retrospectively to our cohort of clinically characterised patients, and of the 55 patients, 30 (55%) patients met the Mayo Clinic HISORt criteria, 31 (56%) patients the Japan Pancreas Society (JPS) criteria, and 45 (82%) patients the Korean criteria. Twenty-seven patients met all three diagnostic criteria; six patients met two diagnostic criteria (4 met JPS and Korean and 2 met HISORt and Korean); and thirteen patients met only one diagnostic criterion (12 met Korean and 1 met HISORt). There were nine patients who were included in the study that did not meet any of the diagnostic criteria and they are discussed later.

When stratified by diagnostic criteria the percentage of males and mean age remained similar amongst all groups (Table 2). There were no differences in presentation, serum biochemistry, liver enzymes or imaging findings. Of note the median serum IgG4 level appeared higher in those that did not meet a diagnostic criteria, suggesting a bias in the approach of clinicians. Similar number of patients were treated with steroids who met the Mayo Clinic HiSORt criteria (21/30, 70%), JPS (22/31, 72%) and Korean Criteria (31/45, 69%)). The response to steroid therapy was seen in similar proportions of patients who met each criterion: HISORt 17/30 (57%), JPS 14/31 (45%), and Korean 23/45 (51%) (Table 3). Patterns of meeting diagnostic criteria were assessed and the majority (HISORt 18/30 (60%), JPS 17/31 (55%), and Korean 26/45 (58%)) met a diagnostic criterion with radiological findings and elevated serum IgG4 levels (Table 4). Only seven patients had histologically proven AIP, a reflection of the clinical practice in Toronto. The proportion of patients meeting typical radiological changes was similar amongst all three diagnostic criteria.

**Table 2:** Percentage of patients meeting diagnostic criteria

**Diagnostic factors** **n = 27 (49%)**	**All criteria met**	**HISORt (2006)** **n = 30, (55%)**	**Japanese (2006)** **n = 31 (56%)**	**Korean (2007)** **n = 45 (82%)**	**No criteria met** **n = 9 (16%)**
**I. Typical imaging**	20 (74%)	21 (70%)	20 (65%)	27 (60%)	4 (44%)
**Ia. Atypical imaging**	7 (26%)	9 (30%)	11 (35%)	18 (40%)	2 (22%)
**II. Lab IgG4**	21 (81%)	23 (77%)	26 (84%)	26 (58%)	3 (33%)
**III. Histology**	7 (30%)	8 (27%)	8 (26%)	8 (18%)	0 (0.0%)
**IV. Other organ involvement**	18 (70%)	22 (73%)	21 (68%)	28 (62%)	2 (22%)
**V. Steroid response**	14 (52%)	17 (57%)	14 (45%)	23 (51%)	1 (11%)
**Definite diagnosis**					
	-	III (8)	I + II (17)	I + II (18)	-
				Ia + II (8)	
	-	I + II (18)	Ia + II (9)	I + III (3)	-
				Ia + III (2)	
	-	Ia + II + V (2)	I + III (3)	I + IV (4)	-
				Ia + IV (2)	
	-	Ia + IV + V (2)	Ia + III (2)	I + V (5)	-
				Ia + V (3)	

**Table 3:** Clinical characteristics of patients suspected of AIP but who did not meet diagnostic criteria

**Patient characteristic**	**PSC and elevated IgG4**	**Radiological changes c/w AIP**
Number of patients	4	5
Males (n, %)	4 (100%)	4 (80%)
Age (years, mean)	63	54
IgG4 (g/L, median)	7.3	NA
IgG4 > 2 × ULN (n, %)	4	
Biochemistry		
Bilirubin (μmol/L*)	11.0 (11–124)	47.0 8–217)
ALP (U/L*)	330.0 (71–1500)	204.0 (100–303)
ALT (U/L*)	62.0 (23–234)	91.0 (29–115)
AST (U/L*)	54.5 (17–229)	41.5 (21–94)
Presentation		
Obstructive jaundice	1	4
Weight loss	2	4
Abdominal pain	2	5
Imaging modality		
CT (n, %)	3	5
MRCP (n, %)	4	5
ERCP (n, %)	2	4
Radiological pancreatic parenchyma typical		
Diffuse	0	4
Multifocal	0	0
Peripheral rim enhancement	1	4
(Halo)	1	1
Atypical		
Focal	0	0
Atrophic		
AIP Radiological classification		
SC	3	0
SP-SC	0	3
SP	0	2
Biliary changes		
Intrahepatic duct	4	2
Proximal extra-hepatic duct	2	4
Initial treatment		
Steroids	3	0
ERCP and biliary stenting	0	4
Steroids and biliary stenting	0	0
Response to steroids	1	0
Complications		
Cholangiocarcinoma	1	0

*Median.

**Table 4:** Response to treatment

	**Normal liver enzymes**	**Improved liver enzymes**	**Stricture resolution**
Steroids (n = 18)	10 (56%)	2 (11%)	3 (17%)
Biliary stent (n = 11)	6 (55%)	2 (18%)	6 (55%)
Biliary stent + steroids (n = 16)	11 (69%)	5 (31%)	8 (50%)

### Patients with suspected disease

There were 9 patients who were suspected clinically to have an IgG4 systemic disease but who did not meet a pre-defined diagnostic criterion for AIP. Two groups were identified: those with sclerosing cholangitis and elevated IgG4 and those with isolated radiological changes consistent with AIP. In those with radiological changes, almost all presented with either abdominal pain (5/5), obstructive jaundice (4/5), or weight loss (4/5) whereas these symptoms were not predominant in those with sclerosing cholangitis and an elevated IgG4. Four of the five patients in the radiological group required ERCP and stenting with complete resolution of symptoms. IgG4 was > 2 × ULN in all patients with possible sclerosing cholangitis and an elevated IgG4 but was not available for review for those with radiological changes. The former group of patients with sclerosing cholangitis and an elevated IgG4 includes one patient who responded to steroids whilst none of the patients in the radiological group were treated with steroids. Histology was not obtained for any patients in this group of suspected of AIP. Extra-pancreatic manifestations were not commonly seen in these patients: one patient in the sclerosing cholangitis and an elevated IgG4 group had IBD and one patient in the radiological group had lymphadenopathy.

## Discussion

In this large Canadian descriptive cohort of patients with autoimmune pancreatitis/IgG4 related disease, we describe the attributes of 55 patients with clinical disease, carefully reviewed from a larger cohort of suspected patients. We provide confirmation of the multisystem nature of disease, the critical role for high quality imaging in reaching a diagnosis, and the response to treatment with steroids, that mandates early diagnosis, so ensuring good outcomes for patients. We also demonstrate the utility of scoring systems that do not focus on histologic evaluation, and therefore are more readily applicable generally to all specialists in the field.

Our clinic series is representative of a general referral practice that captures secondary and tertiary hepatobiliary disease in a large metropolitan city, and as a result provides an insight into managing this condition, particularly where there is a reality, which reflects clinical practice, to focus on radiologic evaluation rather than histology in the diagnostic pathway. When reaching a diagnosis of AIP clinicians are faced with an immediate challenge of deciding what certainty is required to both exclude malignancy and to justify steroids. A majority of the patients presented with either elevated liver enzymes or extra-hepatic biliary strictures and serum IgG4 levels were greater than two times upper limit of normal in slightly over half of the patients. It is thus highly appropriate to consider testing IgG4 at presentation in such patients, as this may facilitate a more rapid diagnosis and possibly avoid interventions such as surgery, if a trial of steroids is contemplated. For the majority of community gastroenterologists and hepatologists it is not usual practice (or necessarily easy) to get tissue diagnosis when managing patients with presentations such as AIP. Unlike serum IgG4 measurements, IgG4 immunostaining may not be a routine immunohistochemistry investigation. In our patient population, many patients were successfully treated for AIP but when compared to the various validated diagnostic criteria, not all would have met criteria in other series. The various scoring systems each originate from different settings, and have an inherent academic bias. We applied these scoring systems to our cohort, and found that the Korean criterion was of greatest utility in our cohort, with 82% of our patients meeting the proposed criteria. This reassures clinicians for whom histology is infrequently obtained, that it is safe to diagnose and treat patients using an approach heavily biased towards IgG4 elevations and imaging.

Our cohort represents a multi-ethnic community served by a relatively few tertiary centres in Toronto, and therefore captures a fair cross-sectional representation of this disease in Canada and North America. We are thus able in this population to confirm the male predominance of disease, with presentation most commonly in those over 50 years of age. This male predominance is of course quite unusual for an autoimmune disease, and whilst the name given to the disease suggests classic autoimmunity, there remains a lack of good pathophysiologic understanding, including in particular the absence of a true autoantigen. Indeed the ethnic mix might be argued to suggest a stronger common environmental stimulus for disease, than a common genetic background.

Much has been discussed about the overlap in terms of imaging of the biliary tree in PSC as compared to classic AIP [18]. The incentive for pursuing such overlap is of course driven by the absence of PSC therapy, as well as the frequent IgG4 elevations seen in patients with PSC [12,13]. Of those patients in our cohort who did not meet classic criteria for AIP, 3 had sclerosing cholangitis with high IgG4. Their response to immunosuppression was not dramatic, and notably they had limited radiologic evidence of more classic AIP. This cautions clinicians about the initial optimism for steroids in a subset of PSC patients with elevated IgG4, and again reiterates the need for prospective research in this area. Additionally, it favours the importance of radiologic features of autoimmune pancreatitis in predicting outcome from steroid treatment.

Retrospective descriptive review is inevitably limited as compared to prospective studies, but for a rare disease managed by many clinicians, across in-patient and out-patient settings, it remains distinctly hard to be prospective in ones approach. Nevertheless clinicians and patients are in need of descriptions of disease that match their own experience and practice, as is the case here: our data therefore adds to the breadth of experience described for this difficult and often enigmatic disease, and highlights how patients have presented and been managed over time, as concepts about the disease have evolved, and clinicians have grown in their experience. Ideally specialist clinics would triage referrals and facilitate more prospective studies to address more closely the role of radiology over histology in diagnosing this syndrome. Clearly our approach can’t formally validate the scoring systems in use (and isn’t designed to; nor is it designed to utilise every scoring system presently available not least because not all clinical information was available to us, and this obstacle could not be overcome retrospectively), but nevertheless we can demonstrate their applied utility, which reflects the interest of clinicians. Similarly our observations largely reflect the type 1 AIP disease spectrum, and there will inevitably be patients presenting with type 2 disease (albeit much less common) who do not adequately fit into our description.

Autoimmune pancreatitis is notably a highly steroid sensitive disease. In our series there was a lower than expected steroid response to treatment. The likely explanation for this, once again, reflects the real world practice described. In settings where patients present to a variety of clinicians, in which referral pathways are set and potentially limited by pressure on resources, it is more likely that patients will have a delay in diagnosis and hence a potential impact on treatment. Additionally without clinical confidence from treating prior patients, clinicians may not always capture diagnosis accurately i.e. there is a risk of over-diagnosis. Our series, and many others, are therefore of importance in repeatedly highlighting this disease once again to a general audience. We demonstrate that it is important to consider autoimmune pancreatitis as a potential diagnosis, and that the combination of prompt high quality radiologic imaging and serologic studies, should allow all clinicians to make the diagnosis, and instigate prompt intervention.

## Conclusion

In conclusion we present a descriptive account of a large cohort of patients with a rare multi-system disease, and in so doing provide clinical insight into the management of autoimmune pancreatitis, supporting in particular the use of a radiologic centred approach to diagnosis and management. This parallels the reality faced by many clinicians not able to access histology for diagnosis, and provides ongoing support for the development of diagnostic pathways that are adaptable to varied clinical settings.

## Abbreviations

AIP: Autoimmune pancreatitis; ERCP: Endoscopic retrograde cholangiopancreatography; IBD: Inflammatory bowel disease; JPS: Japan pancreas society; MRCP: Magnetic resonance cholangiopancreatography; PSC: Primary sclerosing cholangitis; ULN: Upper limit of normal.

## Competing interests

The authors declare that they have no competing interest.

## Authors’ contributions

Dr. Hirschfield acts as guarantor for this paper. GMH, KK, PK and GM initiated the study. HP and EL reviewed and analysed all the data. Radiology review was performed by KK, KTK, and LY. The manuscript was written by HP and GMH, and reviewed by all authors. All authors read and approved the final manuscript.

## Pre-publication history

The pre-publication history for this paper can be accessed here:

http://www.biomedcentral.com/1471-230X/13/168/prepub

## Supplementary Material

Additional file 1: Table S1 Diagnostic Criteria. Click here for file
