# Supplementary material for: IgG4 Related disease – a retrospective descriptive study highlighting Canadian experiences in diagnosis and management
Source: BMC Gastroenterol. 2013 Dec 9;13:168. doi: 10.1186/1471-230X-13-168 (PMC3878912; doi:10.1186/1471-230X-13-168)
Supplement: Additional file 1: Table S1 — Diagnostic Criteria. [file 1471-230X-13-168-S1.docx]

Additional file 1: Table S1.Diagnostic Criteria

| **Diagnostic Factors** | **Mayo clinic HISORt (2006)** | **Japan Pancreas Society (2006)** | **Korean Criteria (2007)** |
| --- | --- | --- | --- |
| **I. Imaging** | -Not Essential  - I. Typical  -Ia. Atypical | -Essential  -Narrowing of the MPD plus enlargement of pancreas  - I. Typical  -Ia. Atypical | -Essential  -same as JPS  - I. Typical  -Ia. Atypical |
| **II. Lab IgG4** | IgG4 | -GGT, IgG, IgG4  -autoantibody | -IgG or IgG4  -autoantibody |
| **III. Histology** | - LPSP  -IgG4+ cells | -LPSP | -LPSP  -IgG4+ cells |
| **IV. Other organ** | -Renal, RP, LN, lung, etc  - Response to steroid | -not included | -Renal, RP, LN, lung, etc  - Response to steroid |
| **V. Steroids Response** | -Pancreatic lesion  -Extrapancreatic lesion | -not included | -Pancreatic lesion  -Extrapancreatic lesion |
| **Definite Diagnosis** | III | I + II | I + II, Ia + II |
|  | I + II | Ia + II | I + III, Ia + III |
|  | Ia + II + V | I + III | I + IV, Ia + IV |
|  | Ia + IV + V | Ia + III | I + V, Ia + V |
